# Diagnosis of multivessel coronary artery disease using ^13^N-ammonia positron emission tomography and contributing factors of reduced global MFR in the real-world clinical practice

**DOI:** 10.1007/s11604-026-01957-z

**Published:** 2026-03-19

**Authors:** Naoto Kawaguchi, Hideki Okayama, Kentaro Ohara, Shinsuke Kido, Kuniaki Hirai, Tomohisa Okada, Megumi Matsuda, Takeshi Inoue, Teruhito Kido

**Affiliations:** 1 Department of Radiology, Ehime University Graduate School of Medicine, Toon City, Ehime Prefecture 791-0295 Japan; 2 Department of Radiology, Ehime Prefectural Central Hospital, Matsuyama, Ehime Japan; 3 Department of Cardiology, Ehime Prefectural Central Hospital, Matsuyama, Ehime Japan

**Keywords:** ^13^N-ammonia PET, Myocardial flow reserve, Myocardial blood flow, Multivessel coronary artery disease

## Abstract

**Purpose:**

Quantitative ^13^N-ammonia positron emission tomography (PET) allows measurement of myocardial blood flow (MBF) and myocardial flow reserve (MFR). A reduced global MFR may indicate multivessel coronary artery disease (MVD), but its specificity is limited in real-world clinical practice. We aimed to classify the causes of reduced global MFR and to identify factors associated with the diagnosis of MVD.

**Materials and methods:**

Among patients who underwent ^13^N-ammonia PET and invasive coronary angiography (ICA) between April 2014 and October 2018, 81 patients with reduced global MFR (< 2.0) were included. Causes of reduced MFR were categorized based on PET, echocardiography, and ICA findings. Patients were stratified into MVD and non-MVD groups and compared in terms of clinical characteristics, cardiovascular risk factors, echocardiography parameters, and PET imaging. Univariate and multivariate analyses were performed to identify independent predictors of MVD.

**Results:**

MVD was identified in 51% of patients, while 49% were classified as non-MVD. Global MFR did not differ significantly between the MVD and non-MVD groups (1.44 ± 0.32 vs. 1.53 ± 0.34; *p* = 0.220). On multivariate analysis, the presence of a perfusion defect with a fill-in pattern on qualitative PET imaging was the only independent predictor of MVD (OR 13.80; *p* = 0.002). Among non-MVD patients, contributors to reduced global MFR included high resting MBF in 22, insufficient pharmacological stress in 12, influence of body motion artifacts in 12, severe single-vessel disease in 7, and heart failure in 5. Multiple contributing factors were suspected in 19 patients. Five patients were classified as “others,” but were suspected of having coronary microvascular dysfunction.

**Conclusions:**

In real-world clinical practice, global MFR can be reduced by multiple factors. Among patients with reduced global MFR, the presence of a perfusion defect with a fill-in pattern on qualitative PET imaging is the strongest indicator of MVD.

**Supplementary Information:**

The online version contains supplementary material available at 10.1007/s11604-026-01957-z.

## Introduction

^13^N-ammonia positron emission tomography (PET) has demonstrated high diagnostic accuracy for ischemic heart disease [1–3]. In particular, quantitative assessment of myocardial perfusion enables the detection of left main or multivessel coronary artery stenosis, which is often challenging to diagnose using myocardial perfusion single photon emission computed tomography (SPECT) [4, 5]. Furthermore, numerous studies have revealed that reduced global myocardial flow reserve (MFR) is associated with adverse cardiovascular outcomes [6–9]. An MFR below approximately 2.0 is generally considered abnormal, and patients with reduced MFR have higher rates of cardiac events; indeed, MFR often emerges as the strongest predictor of major adverse cardiac events in multivariate analyses [6]. However, in real-world practice, reduced global MFR is not specific to multivessel coronary artery disease (MVD), as various physiological and technical factors can decrease measured values even in the absence of obstructive coronary artery disease (CAD).

Accordingly, we aimed to investigate the underlying contributors to reduced global MFR in the real-world clinical practice and to identify key indicators that improve the diagnostic accuracy of ^13^N-ammonia PET for MVD.

## Methods

### Study patients

Between April 2014 and October 2018, 430 consecutive patients with suspected CAD who were referred for ^13^N- ammonia PET at Ehime Prefectural Central Hospital were enrolled in this retrospective study. Of these patients, 154 had a reduced global MFR (< 2.0). The exclusion criteria were as follows: (1) prior coronary artery bypass grafting (CABG), (2) unstable angina, (3) cardiomyopathy, and (4) insufficient data.

Ten patients were excluded based on these criteria (CABG, *n* = 9; insufficient data, *n* = 1). Among the remaining patients, 82 underwent invasive coronary angiography (ICA) within 6 months of ^13^N- ammonia PET, as determined by the attending physician. One patient was excluded because of a cardiovascular event between PET and ICA. Thus, 81 patients in this study (median age, 72 years; 56 men) were included in the final analysis.

The institutional ethics committee approved the study protocol in accordance with the ethical guidelines of the Declaration of Helsinki (Ehime Prefectural Central Hospital, No. 05- 51) and waived the requirement for informed consent due to the retrospective, observational design of the study. An opt-out approach was applied to obtain consent.

### PET acquisition protocol and image analysis

All patients underwent single-day rest/stress ^13^N-ammonia PET using a PET/CT system (Discovery ST Elite; GE Healthcare, Milwaukee, USA). Dynamic PET scans at rest and during the pharmacologic stress were performed in the 2-dimensional list mode, and dynamic frames were reconstructed (12 × 10 s, 4 × 60 s, 2 × 120 s, and 1 × 10 min, for a total of 20 min) [3]. Patients were instructed to fast for more than 6 h and instructed to refrain from consuming beverages containing caffeine for at least 12–24 h before the test. Current smokers were instructed to abstain from smoking for at least 24 h before the test to minimize the acute effects of smoking on myocardial perfusion. Additionally, patients were advised to avoid using coronary vasodilators for at least 24 h before the test. Both rest and stress scans with adenosine triphosphate (ATP) (ADETPHOS-L KOWA INJECTION; Kowa Company, Ltd, Aichi, Japan) disodium-induced hyperemia at a rate of 160 μg kg^−1^ min^−1^ over 5–6 min were performed by using 370 MBq (10 mCi) of ^13^N-ammonia as a bolus injection with saline flushes, at intervals of 1 h or more [10]. Heart rate and blood pressure were recorded at the time of rest test and every minute during and after ATP infusion, with continuous electrocardiographic monitoring. Informed consent was obtained from all patients for the ^13^N-ammonia PET examination, including the use of ATP as the pharmacologic stress agent.

Visual assessment of the PET perfusion images was performed by two experienced readers (N.K. and K.H.). Myocardial perfusion was visually assessed for any stress perfusion defects and their reversibility. A “perfusion defect with a fill-in pattern” was defined as a regional perfusion defect on stress images that partially or completely normalized on rest images (reversible defect). A case was considered positive when a clearly defined fill-in pattern was visually identified in at least one segment (visual summed difference score (SDS) ≥ 1). All assessments were performed on a patient-based basis, and the same images (short-axis, horizontal long-axis, vertical long-axis, and bull’s-eye images from both stress and rest studies) were independently evaluated in a blinded manner. Disagreements were resolved by consensus after discussion. Patients with a history of myocardial infarction (MI) or a fixed perfusion defect in a coronary artery territory on myocardial perfusion images were classified as having prior MI.

Visual splenic switch-off (SSO) was evaluated by visually comparing the uptake of radioactive tracers in the spleen as observed in the PET images [11]. A positive SSO was defined as a clear decrease in the splenic radiotracer uptake from rest to stress, whereas a negative visual SSO was defined as a visually similar splenic radiotracer uptake at rest and during stress. In addition, the splenic response ratio (SRR) was calculated. Standardized uptake values (SUV, Bq g^−1^) of the spleen and liver were obtained at both rest and stress. The spleen-to-liver ratio at rest (SLR_rest) was defined as splenic SUVmean rest / liver SUVmean rest, and the spleen-to-liver ratio at stress (SLR_stress) was defined as splenic SUVmean stress / liver SUVmean stress. Subsequently, the SRR was calculated as SLR_stress / SLR_rest. Patients with an SRR ≤ 0.88 were classified as splenic responders, while those with values above this threshold were classified as non-responders, as described by Saad et al. [12].

### Quantitative PET analysis

Quantitative PET images were analyzed using a commercially available software package (Syngo MBF; Siemens Healthineers, Forchheim, Germany). Quantitative analysis was almost automatic using a two-compartment model for the model previously developed by Hutchins et al. [13]. The global MFR was calculated as the ratio of the global stress myocardial blood flow (MBF) to rest MBF. Reduced MFR was defined as an MFR < 2.0. We did not apply rate–pressure product (RPP) correction in the MFR analysis.

### ICA analysis

Diagnostic ICA was performed according to standard clinical protocols. MVD was defined as either > 50% stenosis of the left main trunk or significant stenoses (> 70% stenosis) in two major coronary arteries including the left anterior descending artery (LAD).

### Reduced MFR analysis

Several factors other than MVD were considered as potential contributors to reduced global MFR. The contributing factors were categorized into “technical factors” and “physiological/pathological factors,” which were further divided into the following subgroups:

 < Technical factors > 

1. Insufficient pharmacological stress: Patients without a significant increase in heart rate during stress (< 10 bpm) or those with a negative SSO sign [11, 12, 14, 15].
2. Influence of body motion artifact: Patients with marked baseline fluctuation in the time-activity curve (TAC) and documented motion during PET acquisition. Marked baseline fluctuation in the TAC refers to a condition where the TAC exhibits irregular and pronounced up-and-down fluctuations during the phase when it should reach a steady state, occurring across multiple coronary artery territories.

 < Physiological/Pathological factors > 

(3) High resting MBF: Patients with a global rest MBF of ≥ 1.1 mL min^−1^ g^−1^ were categorized into this group.
(4) Heart failure: Patients with a left ventricular ejection fraction (LVEF) of ≤ 50% on echocardiography.
(5) Severe single-vessel disease: Patients with severely stenotic single-vessel disease (not multivessel disease), which may contribute to reduced global MFR.
(6) Others: Patients with reduced global MFR not attributable to the above factors, in which coronary microvascular dysfunction (CMD) was suspected to be the cause.

### Statistical analyses

Data are expressed as mean ± standard deviation (SD), median with interquartile range, or number and percentage (%). Student’s *t*-test or Wilcoxon rank-sum test was used to compare the results in this study as appropriate. Univariate analysis was performed to compare patients with MVD and non-MVD groups for the following variables: sex, age, body mass index (BMI), cardiovascular risk factors, chest pain, prior MI and coronary intervention, echocardiographic findings, and ammonia PET results. Multivariate logistic regression analysis was performed to identify predictors of MVD. All statistical analyses were performed using SPSS version 25 (IBM SPSS Statics, Armonk, NY, USA). Statistical significance was set at *p* < 0.05.

## Results

Table 1 summarizes the patients’ clinical characteristics. Based on ICA results, 41 of the 81 patients (51%) had MVD, whereas 40 (49%) had non-MVD. Among the non-MVD group, five patients showed no significant coronary artery stenosis. “A perfusion defect with a fill-in pattern” on qualitative perfusion images was observed in 39 patients (95%) in the MVD group and in 23 patients (58%) in the non-MVD group. Interobserver agreement between the two readers was substantial (*κ* = 0.87, 95% confidence interval [CI], 0.75–0.99).

**Table 1:** Clinical characteristics of the patients

Characteristics	*n* = 81
Sex, man, n (%)	56 (69%)
Age (years)	72 (63–78)
Body weight (kg)	61 (53–65)
Body mass index (kg/m^2^)	23.7 ± 2.7
Risk factors, n (%)	
Hypertension	59 (73%)
Diabetes mellitus	41 (51%)
Hyperlipidemia	31 (38%)
Family history of CAD	26 (32%)
Chronic kidney disease	13 (16%)
Smoking	42 (52%)
Past	36
Current	6
Coronary intervention, n (%)	16 (20%)
Previous myocardial infarction, n (%)	16 (20%)

Data given as mean ± SD, medians and interquartile ranges, or n (%); *CAD * coronary artery disease

### Factors associated with reduced MFR

Among the 40 cases with non-MVD, the following contributing factors were identified: high resting MBF in 22 patients, insufficient pharmacologic stress in 12 (negative visual SSO sign: *n* = 5; insufficient increase in heart rate during stress: *n* = 6; both signs: *n* = 1), influence of body motion artifacts in 12, severe single-vessel CAD in 7, and heart failure in 5 (Table 2). Multiple contributing factors were suspected in 19 patients. Five patients had none of the identified factors and were classified as “others,” including suspected CMD.

**Table 2:** Factors of associated with reduced MFR

Factors	
Multivessel coronary artery disease	41 (51%)
Non-multivessel coronary artery disease	40 (49%)
High resting MBF	22 (55%)
Insufficient pharmacological stress	12 (30%)
Body motion artifacts	12 (30%)
Severe single-vessel coronary disease	7 (23%)
Heart failure	5 (17%)
Others (including CMD)	5 (17%)

Data given as n (%); *MFR* myocardial flow reserve, *MBF* myocardial blood flow, *CMD * coronary microvascular dysfunction

One patient was excluded from the quantitative SSO analysis because the SUV could not be measured. The spleen SUVmean significantly decreased from rest to stress (3.05 [2.73–3.37] vs. 2.20 [1.91–2.39] Bq g^−1^, *p* < 0.01). Supplementary Fig. 1 shows the relationship between visual SSO and SRR. The SRR of positive visual SSO was significantly lower than that of negative visual SSO (0.73 [0.66–0.79] versus 1.00 [0.92–1.18], *p* < 0.01).

**Fig. 1:**
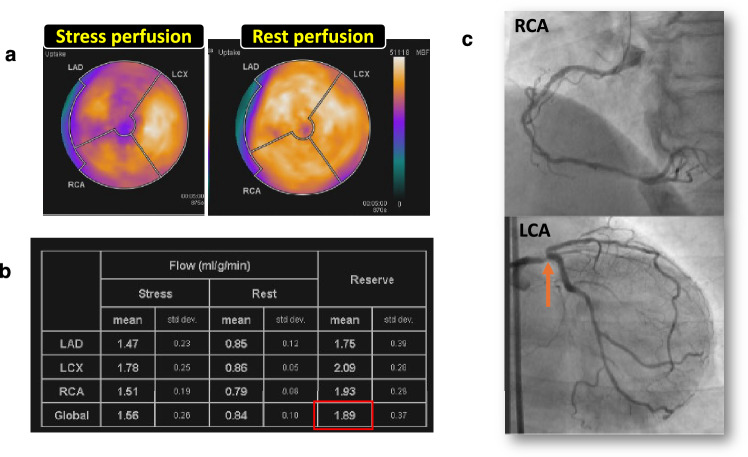
Representative case of MVD. A male patient in his 70 s with hypertension, diabetes mellitus, a past smoker and family history of coronary artery disease presented without chest pain. **a** Qualitative myocardial perfusion image from ammonia PET showing a perfusion defect with a fill-in pattern in the anterior-septal wall region (LAD territory). **b** Quantitative assessment of ammonia PET demonstrating decreased global MFR (< 2.0) (red square line). **c** ICA showing 75% stenosis of the LMT (arrows), 75% stenosis of LAD segments #6–7, and diffuse 50–75% stenosis of the RCA. This case represents a typical presentation of MVD with reduced global MFR and a perfusion defect with a fill-in pattern. *MVD* multivessel coronary artery disease, *PET* positron emission tomography, *MFR* myocardial flow reserve, *LAD* left anterior descending artery, *ICA* invasive coronary angiography, *LMT* left main trunk, *RCA* right coronary artery, *LCA* left coronary artery

**Fig. 2:**
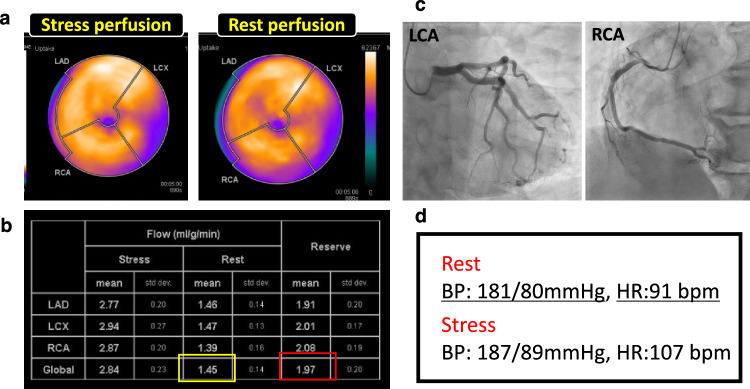
Representative case of high resting MBF. A male patient in his 50 s with hyperlipidemia, a past smoker and a family history of coronary artery disease presented with atypical chest pain. **a** Qualitative myocardial perfusion image from ammonia PET showing no perfusion defect. **b** Quantitative assessment of ammonia PET demonstrating decreased global MFR (< 2.0) (red square line), despite an elevated global rest MBF was high (1.45 mL min^−1^ g^−1^) (yellow square line), **c** ICA showing no significant stenosis. **d** During resting PET acquisition, elevated systolic blood pressure and heart rate resulted in an increased RPP, an elevated resting MBF, and consequently a relative reduction in global MFR. *PET* positron emission tomography, *MFR* myocardial flow reserve, *MBF* myocardial blood flow, *ICA* invasive coronary angiography, *RPP* rate-pressure product, *BP* blood pressure, *HR* heart rate, *LCA* left coronary artery, *RCA* right coronary artery

**Fig. 3:**
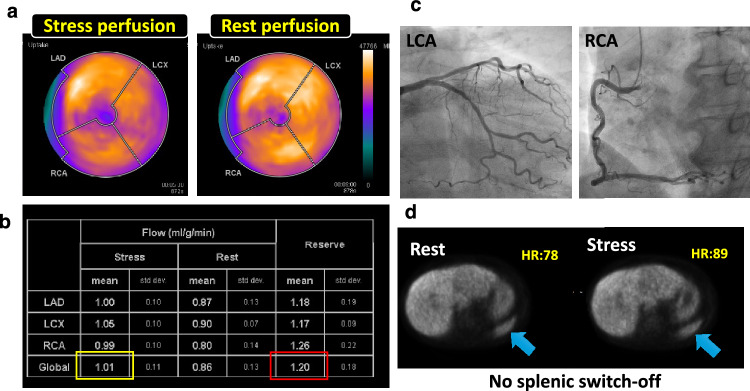
Representative case of insufficient pharmacological stress. A male of patient in his 60 s with hypertension, diabetes mellitus and a past smoker presented without chest pain. **a** Qualitative myocardial perfusion image from ammonia PET showing no perfusion defect. **b** Quantitative assessment of ammonia PET demonstrating significantly decreased global MFR (< 2.0) (red square line), and reduced global stress MBF (1.01 mL min^−1^ g^−1^) (yellow square line). **c** ICA showing no significant stenosis. **d** SSO was negative, indicating an inadequate response to pharmacologic stress. PET positron emission tomography, MFR myocardial flow reserve, MBF myocardial blood flow, ICA invasive coronary angiography, SSO splenic switch-off, HR heart rate, LCA left coronary artery, *RCA* right coronary artery

**Fig. 4:**
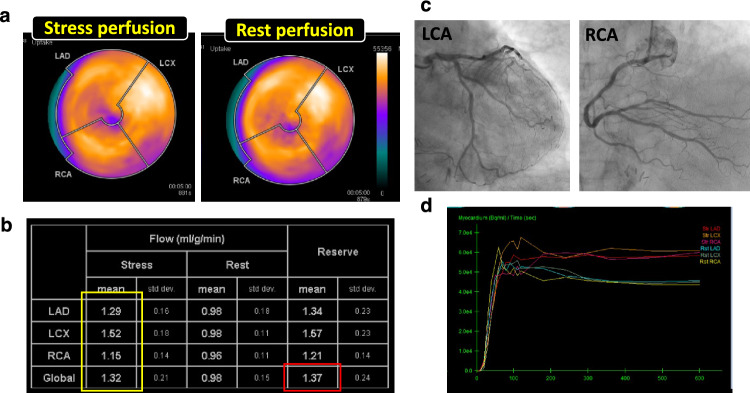
Representative case demonstrating the influence of body motion artifacts. A male patient in his 70 s with hypertension, diabetes mellitus and a current smoker presented without chest pain. **a** Qualitative myocardial perfusion image from ammonia PET showing no perfusion defect. **b** Quantitative assessment of ammonia PET demonstrating significantly reduced global MFR (< 2.0) (red square line) and decreased, heterogeneous global stress MBF across coronary territories (1.32 mL min^−1^ g^−1^) (yellow square line). **c** ICA showing no significant stenosis. **d** The time–activity curves showing large fluctuations during stress attributable due to patient body motion. Although motion correction was applied, substantial motion can render correction incomplete and bias quantitative values. Given the heart rate increase during stress and an LCX-MFR > 1.5, pharmacologic vasodilation was likely adequate. In the absence of an ischemic patterns on relative perfusion images and without significant stenosis on ICA, the heterogeneous reduction in stress MBF is most consistent with motion artifact. However, since the patient has multiple cardiovascular risk factors, a contribution from microvascular dysfunction cannot be excluded. *PET* positron emission tomography, *MFR* myocardial flow reserve, *MBF* myocardial blood flow, *ICA* invasive coronary angiography, *LCA* left coronary artery, *RCA* right coronary artery, *LCX* left circumflex artery

Representative cases are shown for MVD (Fig. 1), high resting MBF (Fig. 2), insufficient pharmacological stress (Fig. 3), and the influence of body motion artifacts (Fig. 4).

### Univariate and multivariate analysis for diagnosing MVD

The characteristics of the patients with and without MVD are summarized in Table 3. There were no significant differences between the two groups in terms of sex, age, BMI, coronary risk factors, chest pain, or presence of SSO. However, echocardiographic findings revealed significant differences in left ventricular end-diastolic volume (median: 86 [71–121] vs. 72 [58–88]), left ventricular end-systolic volume (median: 36 [24–50] vs. 25 [19–35]), and LVEF (median: 61 [55–65] vs. 66 [59–69]). A history of MI and the presence of a perfusion defect with a fill-in pattern on PET perfusion imaging were also significantly more frequent in the MVD group (*p* < 0.05).

**Table 3:** Comparison of patients with MVD and non-MVD

Characteristic	MVD (n = 41)	Non-MVD (n = 40)	p-value (univariate analysis)
Sex, man, n (%)	31 (76%)	25 (63%)	0.202
Age (years)	70 (63–77)	73 (67–78)	0.321
Body mass index (kg/m^2^)	23.9 ± 2.2	23.5 ± 3.2	0.521
Hypertension, n (%)	29 (71%)	30 (75%)	0.666
Diabetes mellitus, n (%)	21 (51%)	20 (50%)	0.913
Hyperlipidemia, n (%)	18 (44%)	13 (33%)	0.291
Smoking, n (%)	18 (44%)	24 (60%)	0.147
Family history of CAD, n (%)	14 (34%)	12 (30%)	0.689
Chronic kidney disease, n (%)	6 (15%)	7 (18%)	0.725
Coronary intervention, n (%)	7 (17%)	9 (23%)	0.540
Chest pain, n (%)	13 (32%)	15 (38%)	0.584
LVEDV (mL)	86 (71–121)	72 (58–88)	0.03
LVESV (mL)	36 (24–50)	25 (19–35)	0.012
LVEF (%)	61 (55–65)	66 (59–69)	0.019
Prior myocardial infarction, n (%)	13 (32%)	3 (8%)	0.006
Perfusion defect with a fill-in pattern, n (%)	39 (95%)	23 (58%)	< 0.0001
Splenic switch-off (negative), n (%)	2 (5%)	6 (15%)	0.127
Global Stress MBF (mL min^−1^ g^−1^)	1.40 ± 0.35	1.76 ± 0.58	0.001
Global Rest MBF (mL min^−1^ g^−1^)	1.00 ± 0.27	1.15 ± 0.28	0.017
Global MFR	1.44 ± 0.32	1.53 ± 0.34	0.220

Data given as mean ± SD, medians and interquartile ranges, or *n* (%); *MVD* multivessel coronary artery disease, *CAD* coronary artery disease, *LVEDV* left ventricular end-diastolic volume, *LVESV* left ventricular end-systolic volume, *LVEF* left ventricular ejection fraction, *MBF* myocardial blood flow, *MFR* myocardial flow reserve

Quantitative PET analysis showed significantly lower global stress MBF (1.40 ± 0.35 vs. 1.76 ± 0.58 mL min^−1^ g^−1^) and global rest MBF (1.00 ± 0.27 vs. 1.15 ± 0.28 mL min^−1^ g^−1^) in the MVD group. Although global stress and rest MBF were significantly lower in the MVD group, global MFR did not differ significantly between groups (1.44 ± 0.32 vs. 1.53 ± 0.34; *p* = 0.220).

Logistic regression analysis was performed to identify independent predictors of MVD (Table 4). Among all variables, the presence of a perfusion defect with a fill-in pattern was the only significant predictor in the model (odds ratio [OR], 13.80; 95% CI, 2.61–72.98; *p* = 0.002). The diagnostic performance of the perfusion defect with a fill-in pattern for MVD was as follows: sensitivity, 95%; specificity, 43%; positive predictive value (PPV), 63%; negative predictive value (NPV), 90%; and overall accuracy, 69%.

**Table 4:** Multivariate predictors for diagnosing MVD

Predictors	Odds ratio (95%CI)	*P*-value
LVEDV (mL)	1.04 (0.97–1.11)	0.275
LVESV (mL)	0.92 (0.80–1.06)	0.238
LVEF (%)	0.91 (0.78–1.08)	0.275
Prior myocardial infarction	5.67 (0.91–35.29)	0.063
Perfusion defect with a fill-in pattern	13.80 (2.61–72.98)	0.002
Global stress MBF (mL min^−1^ g^−1^)	0.21 (0.04–1.08)	0.062
Global rest MBF (mL min^−1^ g^−1^)	2.46 (0.16–37.15)	0.516

*LVEDV* left ventricular end-diastolic volume, *LVESV* left ventricular end-systolic volume, *LVEF* left ventricular ejection fraction, *MBF* myocardial blood flow

## Discussion

This study examined patients in a real-world practice who had reduced global MFR on ^13^N-ammonia PET. Our main findings were as follows:

1. In routine clinical practice, multiple factors can contribute to a reduced global MFR on ammonia PET. In our study, only about half of the patients with MFR < 2.0 actually had multivessel obstructive CAD. These results highlight that a globally reduced MFR does not equate directly with MVD; therefore, careful interpretation is required to identify the underlying cause.
2. The strongest indicator of the patients with MVD in the context of reduced global MFR was a perfusion defect with a fill-in pattern on the qualitative PET perfusion images, as shown by multivariate analysis.

### Patient selection

The primary objective of our study was to determine the prevalence of MVD among patients with reduced MFR who underwent ^13^N-ammonia PET for suspected CAD, and to identify the underlying causes in non-MVD cases. Although prior MI and coronary artery stenosis do not necessarily correspond anatomically, MI cases should ideally be excluded for strict diagnostic accuracy. However, in clinical practice, MVD often includes patients with prior MI (32% in our MVD group); therefore, we did not exclude such cases from our analysis. Notably, qualitative myocardial perfusion findings showing “a perfusion defect with a fill-in pattern” emerged as the strongest predictor of MVD, even in patients with prior MI. This result provides clinically important insight for patients undergoing 13N-ammonia PET for suspected CAD.

### Interpretation of reduced MFR

Quantitative MFR measured by PET has been extensively studied as a diagnostic and prognostic tool [1–9, 17, 18, 19]. A reduced MFR is generally attributed to epicardial coronary artery stenosis or CMD. Previous studies have shown that reduced MFR is associated with multivessel CAD and can uncover ischemia that relative perfusion imaging alone might miss. For instance, Ziadi et al. [4] demonstrated that patients with angiographic triple-vessel disease had significantly lower PET MFR than those without, and a global MFR < 2.0 was an independent predictor of triple-vessel CAD. However, our data strongly support that MFR is sensitive but not specific for obstructive CAD. Many conditions can cause reduced MFR in the absence of any flow-limiting coronary stenosis. Our classification of the major two contributing factors reflects the multifactorial nature of MFR, which can be affected by hemodynamic state (heart rate and blood pressure), endothelial function, extravascular compressive forces, and technical aspects of the PET imaging. In real-world practice, multiple physiological/pathological and technical factors can lead to reduced MFR, and thus a reduced global MFR does not automatically imply the need for revascularization.

### Causes of reduced MFR in patients without MVD

In this study, we classified the potential causes of reduced global MFR in non-MVD cases into two major categories, “technical factors” and “physiological/pathological factors,” and further divided them into six subgroups: (1) insufficient pharmacological stress, (2) influence of body motion artifacts, (3) high resting MBF, (4) heart failure, (5) severe single-vessel disease, and (6) others.

The most frequent was high resting MBF, which was observed in 55% of the non-MVD group. Based on previous literature using the same PET protocol, high resting MBF was defined as ≥ 1.1 mL min^−1^ g^−1^, given that the mean stress MBF in non-stenotic vessels was 2.19 mL min^−1^ g^−1^ [3]. In our cohort, the median rest MBF in the non-MVD group with high resting MBF was 1.34 mL min^−1^ g^−1^, with some values as high as 1.8 mL min^−1^ g^−1^. High resting MBF is not a direct cause of reduced MFR but may rather be considered a phenotypic abnormality. Because high resting MBF can arise from various underlying factors, careful interpretation that considers these factors is required. This condition is often associated with an elevated RPP, which reflects increased resting blood pressure and heart rate. If a patient has a high resting blood pressure or heart rate, the myocardial oxygen demand is elevated and coronary autoregulation will increase resting flow to compensate, thereby reducing the vasodilator reserve. In our study, the RPP was significantly higher in patients with high resting MBF than in those with normal resting MBF group (Supplementary Table 1). Although RPP correction is widely recommended, it may not be appropriate in all cases [20]. The concept of an endogenous phenotype with high resting MBF has also been proposed, and in some cases, may reflect abnormal physiology with CMD [21]. Increases in rest MBF may be influenced by factors such as systemic hypertension, metabolic syndrome, obesity, elevated heart rate, psychological stress or anxiety, female sex, vasodilator or antihypertensive therapy, impaired renal function, hypermetabolic states, or even normal physiological variants associated with preserved microvascular function. In our data, the proportion of female patients and of those not receiving *β*-blockers was significantly higher in the high resting MBF group (Supplementary Table 1). Resting MBF tends to be higher in women, and *β*-blocker therapy, through suppressing heart rate elevation, is associated with lower resting MBF. However, given the limited sample size, further investigation in larger cohorts is required to clarify the determinants of high resting MBF. Taken together, reduced MFR associated with high resting MBF should be regarded as a phenotypic abnormality rather than the result of a single causal factor, and the findings should be interpreted in light of the various underlying factors inherent to this phenotype. Regardless, assessing the RPP is essential when interpreting reduced MFR due to high resting MBF.

Suspect of insufficient pharmacological stress was the second most common cause (30%). Typically, pharmacological stress results in an increase in heart rate and a decrease in blood pressure, and these physiological responses are often used as indicators of adequate vasodilation. In our study, we defined an inadequate stress response as a heart rate increase of less than 10 bpm [16]. However, relying solely on heart rate changes may not be sufficient to assess stress adequacy. The SSO concept has recently attracted attention as an alternative marker [11, 12, 14, 15]. During effective pharmacological vasodilation, splenic perfusion is reduced, and the absence of this reduction—referred to as SSO negativity—has been proposed as an indicator of insufficient stress. We evaluated the presence or absence of SSO using both visual assessment and the SRR. However, it is difficult to determine the appropriate cutoff value for SRR, because the PET modalities, tracers and drugs for pharmacological stress differ from previous studies. In our previous study using SSO at our institution [11], both visual SSO and the SRR were evaluated; however, the final analysis focused on visual SSO. In patients without CAD, global MFR and global stress MBF were significantly higher in the SSO-positive than in the SSO-negative group. The authors concluded that the presence of a splenic response based on visual assessment of SSO may be used to identify an adequate pharmacological response. In the present study as well, based on these considerations, we adopted visual SSO as one of the defining criteria for insufficient pharmacologic stress. However, SSO negativity is not a definitive marker, as false positives can occur. In cases of insufficient stress, stress MBF may not reach maximal hyperemia, either due to a suboptimal pharmacologic response or technical failure, such as inadequate agent delivery or device malfunction. This distinction is critical, particularly in patients with MFR values approaching 1.0, which indicates virtually no increase in MBF from rest to stress. In such cases, careful evaluation of the heart rate response and the presence or absence of SSO may help identify the underlying cause of inadequate vasodilation. Furthermore, stress adequacy should be verified (by repeating the study or performing an alternate stress test) before concluding that the patient has MVD or CMD.

Numerous prior PET studies have highlighted the detrimental impact of patient motion on the accuracy of MBF quantification [22–24]. Motion correction techniques can improve MBF accuracy to some extent; however, substantial patient motion remains a major source of error. Although the Syngo MBF software includes motion correction, large-scale patient movement, particularly during stress, can introduce baseline fluctuations in the TACs and distort kinetic modeling. From a theoretical perspective, motion artifacts lead to underestimation rather than overestimation of MFR. This occurs because frame misalignment during dynamic PET acquisition reduces the apparent myocardial tracer uptake during stress, while images at rest are less affected. Consequently, the stress MBF is underestimated, resulting in a falsely reduced MFR. Our finding that 30% of non-MVD patients had evidence of motion influencing their results underscores the importance of routinely checking TACs or motion correction logs. At our institution, stress operators document significant patient movement, and cases with notable TAC baseline instability were considered to have falsely lowered MFR values due to motion artifacts. Therefore, reviewing the TACs is critical when a global MFR is reduced.

In non-ischemic systolic heart failure, MFR is often reduced even without obstructive coronary disease [25, 26]. Majmudar et al. [25] reported that in patients with non-ischemic heart failure and LVEF ≤ 45%, the median global MFR was 1.67 (1.36–2.08). In patients with heart failure with preserved ejection fraction (LVEF ≥ 50%), MFR is likewise reduced, attributable to abnormal coronary microvascular function and an absolute reduction in the number of resistance vessels of the microcirculation. Thus, even in the absence of obstructive epicardial coronary disease, global MFR can be depressed in patients with heart failure, making MFR alone insufficient for diagnosing MVD.

Severe single-vessel disease: Although the global MFR is typically reduced in MVD, severe stenosis in a single dominant vessel stenosis (e.g., LAD or a dominant left circumflex artery in cases of hypoplastic right coronary artery) may also reduce global MFR. In such cases, the defect is often apparent on visual perfusion images and bull’s-eye maps. However, segmental quantification does not always align perfectly with true coronary territories, so caution is needed [27] .

Others (suspected of CMD): Patients without the above causes classified into the “others” category may have CMD as the underlying cause [28]. Furthermore, even among the patients classified according to the above reasons for reduced MFR, it is presumed that a substantial number of patients had CMD as the primary cause or as a contributing factor. Even in the absence of significant coronary artery stenosis, the presence of typical chest pain symptoms and reduced MFR may suggest the possibility of ischemia with non-obstructive coronary arteries (INOCA) [29]. Such cases typically exhibit diffusely reduced stress MBF and MFR. PET is one of the best noninvasive tools for identifying CMD, as it can demonstrate reduced stress MBF or MFR. Zampella E, et al. and others have shown that such patients have an elevated risk of adverse outcomes despite “normal” coronaries, and addressing their risk factors and symptoms is important [30, 31] . Our data align with this: patients classified as “others (CMD)” likely have diffuse disease and require medical therapy.

Additional tools such as coronary flow capacity (CFC) mapping may help in the diagnosis. CFC is a concept introduced by Johnson et al. [32], which plots absolute stress MBF against coronary flow reserve (CFR) to map the severity and extent of flow impairment. CFC mapping can help differentiate truly normal perfusion, mild diffuse disease, and extensive CMD versus multivessel epicardial disease [33]. Although we did not apply CFC analysis in this study, it represents an advancement in interpretation that integrates relative and absolute data, similar to our approach of using perfusion patterns in conjunction with MFR. Notably, high resting MBF, insufficient stress, and motion artifacts should be excluded before diagnosing CMD.

In a recent report by Pingree et al. [34] identified BMI was identified as the most influential factor affecting MBF and MFR in patients without CAD undergoing quantitative ^13^N-ammonia PET. Additionally, hypertension and coronary artery calcium scores have been reported to influence MFR. Similarly, Sperry et al. [35] demonstrated that in a healthier cohort, both age and sex significantly impacted MBF and MFR measurements using myocardial perfusion PET. Collectively, these findings indicate that the reference values for MFR depend on variables such as age, sex, and BMI, all of which are known to affect the coronary microcirculation. Although the reference ranges for MBF and MFR may vary depending on the PET scanner, the tracer, the analysis method, and the stress protocol, previous studies suggest that an MFR value of < 2 can reasonably be considered abnormal. In the present cohort, all patients underwent both PET and ICA, representing a population with a very high cardiovascular risk. Only five patients (6% of the total) were free of significant coronary artery stenosis. While direct evaluation of CMD remains challenging, appropriately assessing MFR impairment is crucial, especially in high-risk patients without epicardial stenosis, as it plays an essential role in evaluating cardiovascular event risk.

### Identifying MVD in patients with reduced MFR

This study was motivated by the practical challenge of determining when a patient with a reduced MFR actually has multivessel obstructive CAD, as opposed to other causes that can be managed medically. Our multivariate analysis found that of all variables, the presence of a perfusion defect with a fill-in pattern was the only independent predictor of MVD. In contrast, prior MI and low LVEF can indicate CAD but are not specific to multivessel (e.g. a prior MI result from single-vessel occlusion). MVD typically produces regional heterogeneity in perfusion that can be detected by PET, whereas purely diffuse causes tend to depress perfusion uniformly. On SPECT, balanced ischemia can be truly “invisible” due to its limited resolution and reliance on relative uptake, whereas PET, with higher resolution and quantitative flow assessment, can often detect subtle differences [36]. In our study, 95% with MVD patients had at least one regional defect on PET. Essentially, when global MFR is low and a perfusion defect is present, significant CAD should be strongly suspected in that region (and possibly others). In this study, visual assessment of perfusion defects by experts was prioritized over software-derived summed stress or difference scores (SSS/SDS), which may overestimate defect size—especially in apical or basal segments that do not correspond to a coronary territory—or underestimate diffuse disease. Expert reading to identify a true perfusion defect with a fill-in proved to be highly sensitive for CAD. In the present study, we defined a positive fill-in pattern as a clearly delineated fill-in pattern visually identified in at least one segment (visual SDS ≥ 1). This threshold was chosen because the patient cohort exhibited globally reduced MFR, strongly suggesting a high likelihood of MVD. With a threshold of visual SDS ≥ 1, the diagnostic performance for MVD was as follows: sensitivity, 95%; specificity, 43%; PPV, 63%; NPV, 90%; and overall accuracy, 69%. With a threshold of visual SDS ≥ 2, the diagnostic metrics were: sensitivity, 90%; specificity, 43%; PPV, 62%; NPV, 81%; and overall accuracy, 67%. With a threshold of visual SDS ≥ 3, the diagnostic metrics were: sensitivity, 76%; specificity, 55%; PPV, 63%; NPV, 69%; and overall accuracy, 65%. Although the overall accuracy did not differ substantially across thresholds, using SDS ≥ 1 provided substantially higher sensitivity, which is advantageous for minimizing the risk of missed diagnoses of MVD. Moreover, given the high negative predictive value at this threshold, the absence of a perfusion defect may provide a strong basis for considering etiologies other than MVD.

If PET shows no focal perfusion defect despite low MFR, then MVD is less likely (NPV: 90% in our data). Such cases do not necessarily preclude performing ICA. Further testing should focus on alternative etiologies. One approach is to first confirm the adequacy of stress (and, if necessary, repeat PET with a different stressor). If stress is adequate, microvascular ischemia or other non-obstructive factors should be considered. For these patients, medical therapy may be initiated without proceeding to ICA. Additional tests, such as coronary function testing in the catheterization lab (e.g., acetylcholine or Doppler flow wire for CFR), can be performed to confirm CMD, although such testing is not available at all centers. Our data support that when PET perfusion imaging is completely normal (i.e., no reversible defect) despite a low MFR, MVD is unlikely; therefore, unnecessary invasive procedures can often be avoided.

Ziadi et al. [6] emphasized the prognostic significance of reduced MFR (< 2), showing that patients with low global MFR had worse outcomes even when perfusion scans were “normal SSS” by perfusion. This aligns with the concept that CMD, or diffuse atherosclerosis, reflected by low CFR, confers increased cardiovascular risk. In our study, among patients with reduced MFR, the absence of a perfusion defect with a fill in patten was associated with a lower likelihood of MVD; however, we did not directly assess prognosis. Given that high resting MBF and heart failure can also potential CMD, we recommend considering these potential contributors and incorporating additional testing for CMD and INOCA as part of an integrated diagnostic workup when interpreting reduced MFR.

## Limitations

This study has some limitations. First, the sample size was relatively small, and the analysis was retrospective and single-center. Second, all patients underwent ammonia PET and ICA, resulting in a cohort largely composed of high-risk patients with suspected or established ischemic heart disease. This selection bias may have influenced both the prevalence of MVD among patients with reduced global MFR and the distribution of non-MVD contributors. In clinical practice, ICA is not performed in some patients when ammonia PET findings other than quantitative parameters, as well as patient characteristics, suggest a low likelihood of significant CAD. Consequently, the true prevalence of MVD among patients with reduced global MFR may be lower than that observed in our study. In addition, high resting MBF, one of the non-MVD categories, may be present in patients with reduced global MFR but with preserved global stress MBF. In such cases, the stress MBF findings may be prioritized, and ICA may not be performed. Therefore, the actual number of patients with high resting MBF may be greater than that represented in our cohort. Third, our definition of “insufficient stress” was based on hemodynamic response (heart rate elevation) and SSO. While reasonable, this approach does not guarantee that maximal hyperemia was consistently achieved. We did not directly measure plasma adenosine levels or coronary vasodilatory capacity beyond PET parameters, and some patients may have received adequate pharmacologic stress despite being classified as having insufficient stress. Fourth, the CMD classification in this study was hypothetical, as the confirmation of CMD is challenging. In fact, invasive assessments of microvascular function (e.g., coronary flow reserve or microvascular resistance) were not performed in patients without MVD; therefore, CMD could not be formally evaluated, and its prevalence was likely underestimated. Ideally, we intended to include CMD within the category of physiological/pathological factors, but in practice, it could only be classified under “others.” Furthermore, in a high-risk CAD population such as that in our study, even among the non-MVD group, some patients with other contributing factors may still have had reduced MFR due to underlying CMD. Consequently, although the number of cases categorized as “others” was small, this may have further underestimated the true burden of CMD in this population. Future studies with larger, multicenter cohorts and systematic evaluation of microvascular function are warranted to validate these findings and clarify the role of PET-derived MFR in distinguishing CMD from MVD. Fifth, in our study, patients with normal global MFR (≥ 2.0) were not included; therefore, the diagnostic performance for MVD in this subgroup could not be evaluated. However, Naya et al. [37] reported that a normal MFR (> 1.93) can effectively exclude MVD with a high negative predictive value. They also noted that a low MFR (≤ 1.93) does not necessarily indicate obstructive CAD, as it may reflect non-obstructive conditions such as diffuse atherosclerosis or microvascular dysfunction. These findings are consistent with and support the results of our study.

## Conclusion

In real-world clinical practice, global MFR can be reduced by multiple factors. Among patients with reduced global MFR, the presence of a perfusion defect with a fill-in pattern on ^13^N-ammonia PET strongly increases the likelihood of MVD, whereas its absence makes MVD less likely. A reduced global MFR alone is insufficient to diagnose MVD. Instead, an integrated interpretation that accounts for stress adequacy, resting hemodynamics, body motion artifacts, and the potential contribution of CMD is essential for accurate evaluation.

## Supplementary Information

Below is the link to the electronic supplementary material.

Supplementary Material 1

Supplementary Material 2

Supplementary Material 3

## References

[1] Fiechter M, Ghadri JR, Gebhard C, Fuchs TA, Pazhenkottil AP, Nkoulou RN, et al. Diagnostic value of 13N-ammonia myocardial perfusion PET: added value of myocardial flow reserve. J Nucl Med. 2012;53:1230–4. 22776752 10.2967/jnumed.111.101840

[2] Lee JM, Kim CH, Koo BK, Hwang D, Park J, Zhang J, et al. Integrated Myocardial Perfusion Imaging Diagnostics Improve Detection of Functionally Significant Coronary Artery Stenosis by 13N-ammonia Positron Emission Tomography. Circ Cardiovasc Imaging. 2016;9(116):004768. 10.1161/CIRCIMAGING.116.004768. 10.1161/CIRCIMAGING.116.004768 27609817

[3] Kawaguchi N, Okayama H, Kawamura G, Shigematsu T, Takahashi T, Kawada Y, et al. Clinical Usefulness of Coronary Flow Reserve Ratio for the Detection of Significant Coronary Artery Disease on (13)N-Ammonia Positron Emission Tomography. Circ J. 2018;82:486–93. 28954967 10.1253/circj.CJ-17-0745

[4] Ziadi MC, Dekemp RA, Williams K, Guo A, Renaud JM, Chow BJ, et al. Does quantification of myocardial flow reserve using rubidium-82 positron emission tomography facilitate detection of multivessel coronary artery disease? J Nucl Cardiol. 2012;19:670–80. 22415819 10.1007/s12350-011-9506-5

[5] Danad I, Raijmakers PG, Driessen RS, Leipsic J, Raju R, Naoum C, et al. Comparison of Coronary CT Angiography, SPECT, PET, and Hybrid Imaging for Diagnosis of Ischemic Heart Disease Determined by Fractional Flow Reserve. JAMA Cardiol. 2017;2:1100–7. 28813561 10.1001/jamacardio.2017.2471 PMC5710451

[6] Ziadi MC, Dekemp RA, Williams KA, Guo A, Chow BJ, Renaud JM, et al. Impaired myocardial flow reserve on rubidium-82 positron emission tomography imaging predicts adverse outcomes in patients assessed for myocardial ischemia. J Am Coll Cardiol. 2011;58:740–8. 21816311 10.1016/j.jacc.2011.01.065

[7] Naya M, Tamaki N, Tsutsui H. Coronary flow reserve estimated by positron emission tomography to diagnose significant coronary artery disease and predict cardiac events. Circ J. 2015;79:15–23. 25744627 10.1253/circj.CJ-14-1060

[8] Juarez-Orozco LE, Tio RA, Alexanderson E, Dweck M, Vliegenthart R, El Moumni M, et al. Quantitative myocardial perfusion evaluation with positron emission tomography and the risk of cardiovascular events in patients with coronary artery disease: a systematic review of prognostic studies. Eur Heart J Cardiovasc Imaging. 2018;19:1179–87. 29293983 10.1093/ehjci/jex331 PMC6148746

[9] von Felten E, Benz DC, Benetos G, Baehler J, Patriki D, Rampidis GP, et al. Prognostic value of regional myocardial flow reserve derived from (13)N-ammonia positron emission tomography in patients with suspected coronary artery disease. Eur J Nucl Med Mol Imaging. 2021;49:311–20. 34191100 10.1007/s00259-021-05459-0 PMC8712296

[10] Miyagawa M, Kumano S, Sekiya M, Watanabe K, Akutzu H, Imachi T, et al. Thallium-201 myocardial tomography with intravenous infusion of adenosine triphosphate in diagnosis of coronary artery disease. J Am Coll Cardiol. 1995;26:1196–201. 7594032 10.1016/0735-1097(95)00304-5

[11] Ohara K, Kawaguchi N, Okayama H, Ueda H, Ueno R, Hirai K, et al. Clinical significance of splenic switch-off in adenosine triphosphate (13)N-ammonia positron emission tomography in patients without coronary artery disease. Jpn J Radiol. 2025;43:1186–96. 40156740 10.1007/s11604-025-01762-0 PMC12204888

[12] Saad JM, Ahmed AI, Han Y, El Nihum LI, Alahdab F, Nabi F, et al. Splenic switch-off in regadenoson (82)Rb-PET myocardial perfusion imaging: assessment of clinical utility. J Nucl Cardiol. 2023;30:1484–96. 36607537 10.1007/s12350-022-03158-3

[13] Hutchins GD, Schwaiger M, Rosenspire KC, Krivokapich J, Schelbert H, Kuhl DE. Non-invasive quantification of regional myocardial blood flow in the human heart using [^13^N] ammonia and dynamic positron emission tomography imaging. J Am Coll Cardiol. 1990;15:1032–342. 2312957 10.1016/0735-1097(90)90237-j

[14] Bakula A, Patriki D, von Felten E, Benetos G, Sustar A, Benz DC, et al. Splenic switch-off as a novel marker for adenosine response in nitrogen-13 ammonia PET myocardial perfusion imaging: cross-validation against CMR using a hybrid PET/MR device. J Nucl Cardiol. 2022;29:1205–14. 33354759 10.1007/s12350-020-02448-y PMC9163112

[15] Patriki D, von Felten E, Bakula A, Giannopoulos AA, Kamani CH, Schwyzer M, et al. Splenic switch-off as a predictor for coronary adenosine response: validation against 13N-ammonia during co-injection myocardial perfusion imaging on a hybrid PET/CMR scanner. J Cardiovasc Magn Reson. 2021;23:3. 33407586 10.1186/s12968-020-00696-y PMC7789581

[16] Karamitsos T, Ntusi N, Francis J, Holloway C, Myerson S, Neubauer S. Feasibility and safety of high-dose adenosine perfusion cardiovascular magnetic resonance. J Cardiovasc Magn Reson. 2010;12:66. 10.1186/1532-429X-12-66. 21080924 10.1186/1532-429X-12-66 PMC2996376

[17] Danad I, Uusitalo V, Kero T, Saraste A, Raijmakers PG, Lammertsma AA, et al. Quantitative assessment of myocardial perfusion in the detection of significant coronary artery disease: cutoff values and diagnostic accuracy of quantitative [(15)O]H2O PET imaging. J Am Coll Cardiol. 2014;64:1464–75. 25277618 10.1016/j.jacc.2014.05.069

[18] Gould KL, Johnson NP, Bateman TM, Beanlands RS, Bengel FM, Bober R, et al. Anatomic versus physiologic assessment of coronary artery disease. Role of coronary flow reserve, fractional flow reserve, and positron emission tomography imaging in revascularization decision-making. J Am Coll Cardiol. 2013;62:1639–53. 23954338 10.1016/j.jacc.2013.07.076

[19] Dietz M, Kamani CH, Allenbach G, Rubimbura V, Fournier S, Dunet V, et al. Comparison of the prognostic value of impaired stress myocardial blood flow, myocardial flow reserve, and myocardial flow capacity on low-dose Rubidium-82 SiPM PET/CT. J Nucl Cardiol. 2023;30:1385–95. 36574175 10.1007/s12350-022-03155-6 PMC10371877

[20] Huck DM, Weber BN, Brown JM, Lopez D, Hainer J, Blankstein R, et al. Prognostic value of myocardial flow reserve vs corrected myocardial flow reserve in patients without obstructive coronary artery disease. J Nucl Cardiol. 2024;37:1. 10.1016/j.nuclcard.2024.101854. 10.1016/j.nuclcard.2024.101854 PMC11257809 38606610

[21] Schindler TH, Fearon WF, Pelletier-Galarneau M, Ambrosio G, Sechtem U, Ruddy TD, et al. Myocardial Perfusion PET for the Detection and Reporting of Coronary Microvascular Dysfunction: A JACC: Cardiovascular Imaging Expert Panel Statement. JACC Cardiovasc Imaging. 2023;16:536–48. 36881418 10.1016/j.jcmg.2022.12.015

[22] Hunter CRRN, Klein R, Beanlands RS, deKemp RA. Patient motion effects on the quantification of regional myocardial blood flow with dynamic PET imaging. Med Phys. 2016;43:1829–40. 27036580 10.1118/1.4943565

[23] Hunter CRRN, Klein R, Alessio AM, deKemp RA. Patient body motion correction for dynamic cardiac PET-CT by attenuation-emission alignment according to projection consistency conditions. Med Phys. 2019;46:1697–706. 30710381 10.1002/mp.13419 PMC9559704

[24] Lee BC, Moody JB, Poitrasson-Riviere A, Melvin AC, Weinberg RL, Corbett JR, et al. Blood pool and tissue phase patient motion effects on (82)rubidium PET myocardial blood flow quantification. J Nucl Cardiol. 2019;26:1918–29. 29572594 10.1007/s12350-018-1256-1 PMC6151305

[25] Majmudar MD, Murthy VL, Shah RV, Kolli S, Mousavi N, Foster CR, et al. Quantification of coronary flow reserve in patients with ischaemic and non-ischaemic cardiomyopathy and its association with clinical outcomes. Eur Heart J Cardiovasc Imaging. 2015;16:900–9. 25719181 10.1093/ehjci/jev012 PMC4592320

[26] Srivaratharajah K, Coutinho T, deKemp R, Liu P, Haddad H, Stadnick E, et al. Reduced myocardial flow in heart failure patients with preserved ejection fraction. Circ Heart Fail. 2016. 10.1161/CIRCHEARTFAILURE.115.002562. 27413034 10.1161/CIRCHEARTFAILURE.115.002562

[27] Pereztol-Valdes O, Candell-Riera J, Santana-Boado C, Angel J, Aguade-Bruix S, Castell-Conesa J, et al. Correspondence between left ventricular 17 myocardial segments and coronary arteries. Eur Heart J. 2005;26:2637–43. 16183694 10.1093/eurheartj/ehi496

[28] Taqueti VR, Di Carli MF. Coronary microvascular disease pathogenic mechanisms and therapeutic options: JACC state-of-the-art review. J Am Coll Cardiol. 2018;72:2625–41. 30466521 10.1016/j.jacc.2018.09.042 PMC6296779

[29] Gulati M, Levy PD, Mukherjee D, Amsterdam E, Bhatt DL, Birtcher KK, et al. 2021 AHA/ACC/ASE/CHEST/SAEM/SCCT/SCMR Guideline for the Evaluation and Diagnosis of Chest Pain: A Report of the American College of Cardiology/American Heart Association Joint Committee on Clinical Practice Guidelines. J Am Coll Cardiol. 2021;78:e187–285. 34756653 10.1016/j.jacc.2021.07.053

[30] Zampella E, Mannarino T, D’Antonio A, Assante R, Gaudieri V, Buongiorno P, et al. Prediction of outcome by (82)Rb PET/CT in patients with ischemia and nonobstructive coronary arteries. J Nucl Cardiol. 2023;30:1110–7. 36352083 10.1007/s12350-022-03144-9

[31] Al-Mallah MH, Nayfeh M, Alrifai M. The role of cardiac PET in diagnosis and prognosis of patients with ischemia with no obstructive coronary arteries (INOCA). Am Heart J Plus Cardiol Res Pract. 2024. 10.1016/j.ahjo.2024.100399. 10.1016/j.ahjo.2024.100399 PMC11141139 38828445

[32] Johnson NP, Gould KL. Integrating noninvasive absolute flow, coronary flow reserve, and ischemic thresholds into a comprehensive map of physiological severity. JACC Cardiovasc Imaging. 2012;5:430–40. 22498334 10.1016/j.jcmg.2011.12.014

[33] van de Hoef TP, Echavarria-Pinto M, Escaned J, Piek JJ. Coronary flow capacity: concept, promises, and challenges. Int J Cardiovasc Imaging. 2017;33:1033–9. 28353034 10.1007/s10554-017-1125-z PMC5489577

[34] Pingree R, Markendorf S, Moysidis D, Ryffel C, Stuetz M, Ghenzi R, et al. Myocardial blood flow reference values for 13N-ammonia PET myocardial perfusion imaging in patients without flow-limiting coronary artery disease. Eur J Nucl Med Mol Imaging. 2025;52:3353–63. 40082264 10.1007/s00259-025-07196-0 PMC12222243

[35] Sperry BW, Metzinger MP, Ibrahim AO, Thompson RC, Cho YJ, Jones PG, et al. Age- and sex-specific myocardial blood flow values in patients without coronary atherosclerosis on Rb-82 PET myocardial perfusion imaging. Circ Cardiovasc Imaging. 2024;17:e016577. 10.1161/CIRCIMAGING.124.016577. 39012951 10.1161/CIRCIMAGING.124.016577

[36] Schindler TH, Schelbert HR, Quercioli A, Dilsizian V. Cardiac PET imaging for the detection and monitoring of coronary artery disease and microvascular health. JACC Cardiovasc Imaging. 2010;3:623–40. 20541718 10.1016/j.jcmg.2010.04.007

[37] Naya M, Murthy VL, Taqueti VR, Foster CR, Klein J, Garber M, et al. Preserved coronary flow reserve effectively excludes high-risk coronary artery disease on angiography. J Nucl Med. 2014;55:248–55. 24408896 10.2967/jnumed.113.121442 PMC3962818

