# Supplementary material for: Diagnosis of multivessel coronary artery disease using 13N-ammonia positron emission tomography and contributing factors of reduced global MFR in the real-world clinical practice
Source: Jpn J Radiol. 2026 Mar 19;44(8):1438–49. doi: 10.1007/s11604-026-01957-z (PMC13400682; doi:10.1007/s11604-026-01957-z)
Supplement: Supplementary file 1 — Supplementary Material 1 [file 11604_2026_1957_MOESM1_ESM.docx]

**Supplemental data**

**Supplementary Table１. RPP and corrected global MFR of the patients with non-MVD**

|  | High resting MBF  (n = 21) | Normal resting MBF  (n = 18) | P-value |
| --- | --- | --- | --- |
| Heart rate during rest (bpm) | 71 (61–84) | 62 (60–66) | 0.088 |
| Systolic blood pressure during rest (mmHg) | 156 (138–176) | 150 (137–157) | 0.379 |
| RPP (mmHg·bpm) | 11988 (9882–13013) | 9391(7694–11330) | 0.017 |
| Global rest MBF (mL·min^-1^·g^-1^) | 1.34 (1.19–1.45) | 0.88 (0.81–1.02) | < 0.0001 |
| Corrected global rest MBF  (ml·min^-1^·g^-1^·mmHg^-1^·bpm^-1^·10^4^) | 1.12 (1.07–1.32) | 0.99 (0.75–1.15) | 0.020 |
| Corrected global MFR | 1.77 (1.61–2.09) | 1.38 (1.00–1.63) | 0.003 |
| Sex, woman, n (%) | 12 (57%) | 3 (17%) | 0.019 |
| Age (years) | 75 (72–78) | 70 (63–77) | 0.148 |
| Body mass index (kg/m^2^) | 24.0 (22.2-24.7) | 23.0 (21.5-26.0) | 0.922 |
| Hypertension, n (%) | 14 (67%) | 15 (83%) | 0.290 |
| Diabetes mellitus, n (%) | 9 (43%) | 10 (56%) | 0.527 |
| Hyperlipidemia, n (%) | 8 (38%) | 5 (28%) | 0.734 |
| Smoking, n (%) | 11 (52%) | 12 (67%) | 0.516 |
| Family history of CAD, n (%) | 6 (29%) | 5 (28%) | 1.000 |
| Chronic kidney disease, n (%) | 5 (24%) | 1 (6%) | 0.190 |
| Medication, n (%) |  |  |  |
| Statin | 15 (71%) | 8 (44%) | 0.112 |
| *β*-blocker | 3 (14%) | 9 (50%) | 0.035 |
| Calcium-channel blocker | 13 (62%) | 12 (67%) | 1.000 |
| ACEI/ARB | 14 (67%) | 10 (56%) | 0.525 |
| Aspirin | 7 (33%) | 9 (50%) | 0.342 |

One patient from the group of high resting MBF was excluded from the analysis due to missing systolic blood pressure.

Data given as medians and interquartile ranges

*RPP* rate-pressure product, *bpm* beat per minute; *MFR* myocardial flow reserve; *MVD*, multivessel coronary disease; *MBF* myocardial blood flow; *CAD*, coronary artery disease; *ACEI*, angiotensin-converting enzyme inhibitor; *ARB*, angiotensin-receptor blocker
