# Supplementary figures and images for: Diagnosis of multivessel coronary artery disease using 13N-ammonia positron emission tomography and contributing factors of reduced global MFR in the real-world clinical practice
Source: Jpn J Radiol. 2026 Mar 19;44(8):1438–49. doi: 10.1007/s11604-026-01957-z (PMC13400682; doi:10.1007/s11604-026-01957-z)

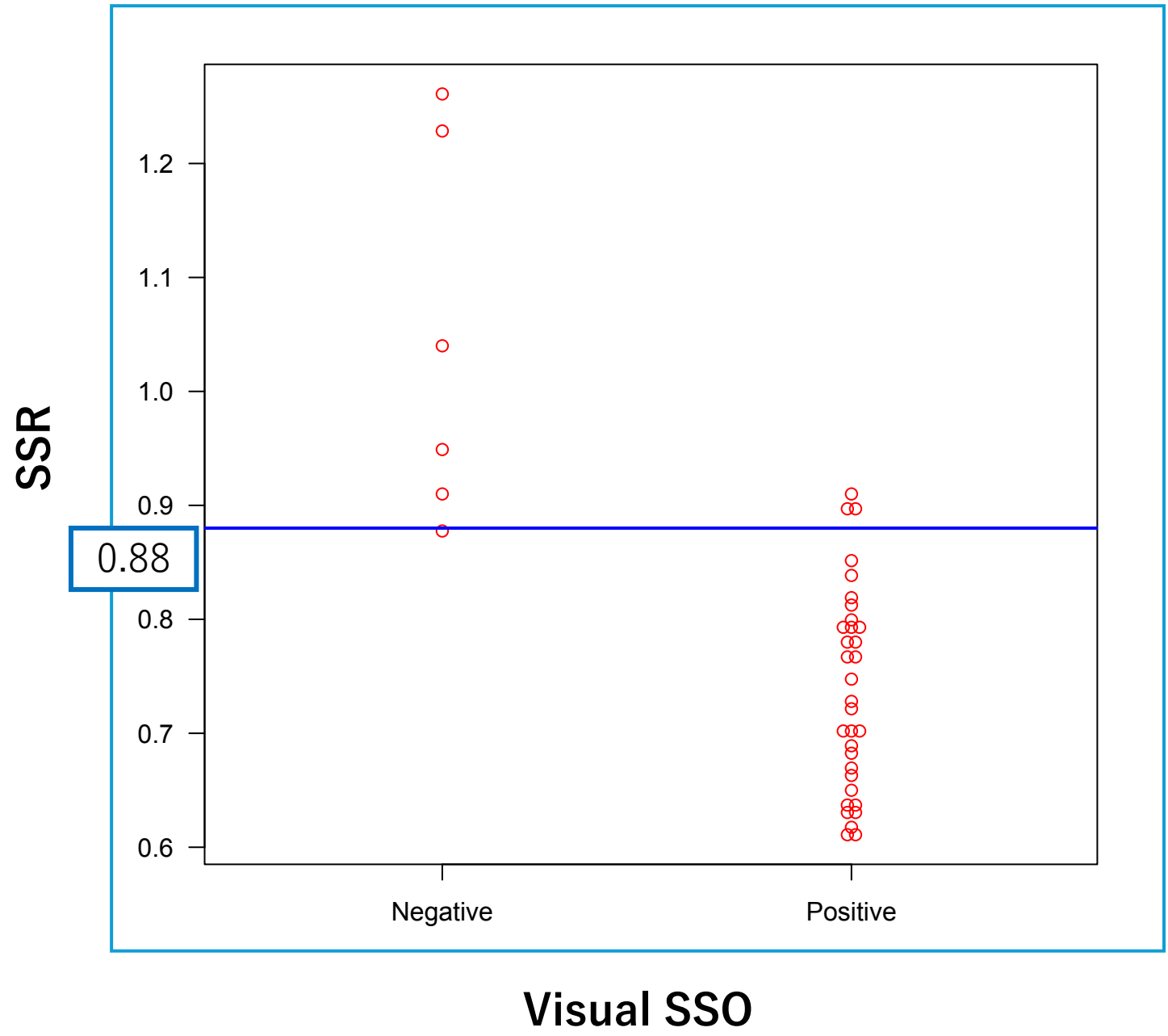

Supplement: Supplementary file 2 — Supplementary Material 2 [file 11604_2026_1957_MOESM2_ESM.pdf]
