# Supplementary material for: Diagnosis of multivessel coronary artery disease using 13N-ammonia positron emission tomography and contributing factors of reduced global MFR in the real-world clinical practice
Source: Jpn J Radiol. 2026 Mar 19;44(8):1438–49. doi: 10.1007/s11604-026-01957-z (PMC13400682; doi:10.1007/s11604-026-01957-z)
Supplement: Supplementary file 3 — Supplementary Material 3 [file 11604_2026_1957_MOESM3_ESM.docx]

Supplementary Figure 1

The relationship between visual SSO and SRR. The SRR of positive SSO was significantly lower than that of negative SSO (median, 0.73 [0.66–0.79] vs. 1.00 [0.92–1.18], p < 0.01). The cutoff line for SRR is shown at 0.88. SSO splenic switch-off, SRR splenic response ratio
